# Multi-band FMRI compromises detection of mesolimbic reward responses

**DOI:** 10.1016/j.neuroimage.2021.118617

**Published:** 2021-09-29

**Authors:** Tara Srirangarajan, Leili Mortazavi, Tiago Bortolini, Jorge Moll, Brian Knutson

**Affiliations:** a Department of Psychology, Stanford University, Stanford, CA, United States; b D’Or Institute for Research and Education (IDOR), Rio de Janeiro, Brazil

**Keywords:** Multi-band, Functional magnetic resonance imaging, Monetary incentive delay, Reward, Anticipation, Accumbens, Frontal, Human

## Abstract

Recent innovations in Functional Magnetic Resonance Imaging (FMRI) have sped data collection by enabling simultaneous scans of neural activity in multiple brain locations, but have these innovations come at a cost? In a meta-analysis and preregistered direct comparison of original data, we examined whether acquiring FMRI data with multi-band versus single-band scanning protocols might compromise detection of mesolimbic activity during reward processing. Meta-analytic results (*n* = 44 studies; cumulative *n* = 5005 subjects) indicated that relative to single-band scans, multi-band scans showed significantly decreased effect sizes for reward anticipation in the Nucleus Accumbens (NAcc) by more than half. Direct within-subject comparison of single-band versus multi-band scanning data (multi-band factors = 4 and 8; *n* = 12 subjects) acquired during repeated administration of the Monetary Incentive Delay task indicated that reductions in temporal signal-to-noise ratio could account for compromised detection of task-related responses in mesolimbic regions (i.e., the NAcc). Together, these findings imply that researchers should opt for single-band over multi-band scanning protocols when probing mesolimbic responses with FMRI. The findings also have implications for inferring mesolimbic activity during related tasks and rest, for summarizing historical results, and for using neuroimaging data to track individual differences in reward-related brain activity.

## 1. Introduction

During the first two decades of the twenty-first century, Functional Magnetic Resonance Imaging (FMRI) revolutionized cognitive neuroscience (Poldrack, 2012). For the first time, researchers could acquire measures of whole brain neural activity (in the form of Blood Oxygen Level Dependent or BOLD activity) at a spatial scale of millimeters and a temporal scale of seconds. This allowed investigators to probe not only neural correlates of sensory and motor processing in living humans, but also diverse intermediate brain processes related to thought and emotion (Rosen and Savoy, 2012). With respect to emotion, FMRI evidence now supports a substantial literature indicating that subcortical circuits play a central role in human reward processing for basic rewards (e.g., juice) as well as more abstract rewards (e.g., money). Different subregions of these neural circuits respond to reward anticipation as well as receipt (Knutson and Cooper, 2005). Using parametric probe tasks, researchers have localized neural activity that scales not only as a function of sensorimotor stimulation, but also as a function of reward magnitude and probability (Knutson et al., 2005; Yacubian et al., 2006). Meta-analyses of hundreds of FMRI studies now indicate that while reward processing engages diverse circuits, the ventral striatum including the Nucleus Accumbens (NAcc) as well as the Medial PreFrontal Cortex (MPFC), are among those regions most likely to show correlated activity (Bartra et al., 2013; Clithero and Rangel, 2014; Knutson and Greer, 2008).

More recently, however, researchers have raised concerns about the robustness and replicability of task-related FMRI findings. These concerns have focused on a number of issues including inadequate power (Button et al., 2013), statistical inflation (Vul et al., 2009), temporal instability (Elliott et al., 2020), and analytic inconsistency (Botvinik-Nezer et al., 2020). Although these issues might have arisen from problems related to study design, analysis, or interpretation, more technical factors related to data acquisition have concurrently changed but have received less scrutiny. For instance, to acquire data more rapidly, researchers have begun to scan brain images simultaneously at different locations (i.e., using multi-band simultaneous slice excitation protocols, hereafter referred to as “multi-band” scans), rather than sequentially and individually (i.e., using single-band individual slice excitation protocols, hereafter referred to as “single-band” scans; Moeller et al., 2010). Multi-band scans confer a significant benefit of yielding the same amount of data in a fraction of the time required by single-band scans. Multi-band scans may, however, incur hidden costs. Despite not diminishing signal-to-noise ratio in cortical regions, multi-band scanning may induce noise in subcortical regions (Todd et al., 2017). For instance, multi-band scanning can substantially increase noise in the subcortical activity of individuals at rest (Risk et al., 2018, 2021), as well as during execution of sensorimotor and cognitive tasks (Demetriou et al., 2018; Todd et al., 2017).

Since reward responses robustly recruit subcortical as well as cortical circuits, we sought to determine whether multi-band versus single-band scanning might systematically influence FMRI detection of neural responses to reward. To do so, we first meta-analyzed the past decade of FMRI findings (i.e., from 2010 to 2020) elicited by a reliable and valid probe of reward processing: the Monetary Incentive Delay or MID task (Knutson et al., 2001; Wu et al., 2014). We specifically aimed to compare responses in the NAcc during reward anticipation and in the MPFC to reward outcomes across studies utilizing single-band versus multi-band scanning protocols.

Next, to reproduce and diagnose potential problems while controlling for possible confounds, we acquired and directly compared MID task data from the same subjects, scanner, and site. Scans used a common protocol which varied only with respect to the speed of image acquisition, coverage, and associated flip angle (i.e., with Multi-Band factors: MB = 1, 4, or 8; Table 1). Analyses focused on raw averaged neural activity extracted from NAcc and MPFC volumes of interest as well as on modeled activity across the entire brain. Based on previous resting state (Risk et al., 2018) and task-related (Demetriou et al., 2018; Todd et al., 2017) findings, we predicted that multi-band versus single-band scanning might compromise detection of reward-related FMRI activity by inducing noise near the center of the brain.

## 2. Methods

### 2.1. Meta-analysis of historical data

To survey the neuroimaging literature for historical evidence that multi-band scanning might have compromised detection of reward-related responses, we conducted a meta-analysis of Volume Of Interest (VOI) FMRI data collected as subjects participated in the MID task. Data came from literature searches using Google Scholar and PubMed for studies published between 2010 and 2020 that used the terms “Monetary Incentive Delay Task AND FMRI”. Single-band and multi-band FMRI studies of healthy adults and/or adolescents were selected if they contrasted gain versus nongain anticipation, and a subset of these studies also contrasted gain versus nongain outcomes. In studies with both healthy and clinical samples, only peak activation values from the healthy samples were included in the analysis. Key exclusion criteria included unpublished preprints (unless the number of subjects exceeded 100), studies that included subjects under twelve years old, functional connectivity studies, and studies using dynamic tasks that required updating of expectations (Table S1 lists excluded studies along with reasons for exclusion). The meta-analysis focused on whether detection of neural responses during gain anticipation in the Nucleus Accumbens (NAcc) and gain outcomes in the Medial PreFrontal Cortex (MPFC) was compromised in studies using multi-band versus single-band scanning.

For meta-analysis, forest plots facilitated direct comparison of reward-related responses (i.e., effect sizes (Cohen’s d) and standard errors) for single-band versus multi-band studies (Radua et al., 2015; Viechtbauer, 2010). Funnel plots then probed for publication bias across these subsets of studies (Egger et al., 1997) using a fixed effects model (R package *‘metafor*; Viechtbauer, 2010). Finally, other variables that correlated with reward-related responses were identified (i.e., subject age, publication date, sample size, flip angle) and included in a multivariate ANalysis Of COVAriance (ANCOVA) to verify the independence and robustness of single-band versus multi-band scanning as a predictor of neural responses to reward.

### 2.2. Direct comparison of original data

Following meta-analysis, we sought to reproduce and diagnose potential problems by acquiring and comparing data using three scanning protocols that varied with respect to multi-band factor (i.e., MB1, MB4, MB8; Table 1). Analyses aimed to localize potential problems in space (i.e., neural location) as well as in time (i.e., during the task versus throughout the entire scan). To control for order effects, subjects completed three runs of the MID task, with the order of the three multi-band factor scans counterbalanced across subjects. To control for subject, scanner, and site, each subject completed all three scan protocols within the same session. All data were preprocessed and analyzed using the same pipelines. The study was approved by the Institutional Review Board of the Stanford University School of Medicine, and all subjects provided written informed consent prior to participating in the study.

#### 2.2.1. Subjects

Preregistered power analysis indicated that for a within-subject comparison, 6 subjects should yield adequate power to detect an effect of multi-band acquisition on the gain versus nongain anticipation contrast in the NAcc during the MID task (g*power 3.1.9.2; beta=0.95, alpha=0.05, two-tailed, paired, *d* = 1.97; Wu et al., 2014). As planned in the preregistration, we doubled this sample size to guard against the possibility of a smaller effect size (*n* = 12; between-subjects). Nineteen subjects completed the MID task while being scanned with a multi-band acquisition protocol. According to the preregistered exclusion criteria, data from three subjects were excluded due to excessive motion during at least one of the three task runs, while data from four subjects were excluded due to equipment failure (i.e., faulty response registration by a new button box), leaving twelve subjects total for analyses.

#### 2.2.2. Task

The MID task was identical across all three runs. The six task trial conditions included: + $5.00 (‘large gain’); +$1.00 (‘medium gain’); +$0.00 (‘nongain’); −$5.00 (‘large loss’); −$1.00 (‘medium loss’); and −$0.00 (‘nonloss’) trials. Each trial condition was repeated 12 times in a pseudorandom order, totaling 72 trials. Trial timing was as follows: cue presentation (seconds 0–2); anticipatory fixation (seconds 2–4); target presentation (appearing briefly between seconds 4–4.5); outcome presentation (seconds 6–8); and a variable Inter-Trial Interval (ITI lasting 2, 4, or 6 s). Thus, each trial lasted an average of 12 s (including the ITI). Adaptive timing of target duration within condition ensured that subjects succeeded in “hitting” targets on approximately 66% of the trials (Knutson et al., 2005). Thus, each MID task run lasted 864 total seconds (approximately 14.4 min), and all three runs were acquired during a single session, but with counterbalanced ordering across subjects.

#### 2.2.3. FMRI acquisition

All data were acquired on a 3 Tesla General Electric scanner with a 32-channel head coil at the Stanford Center for Cognitive and Neurobiological Imaging (CNI). Structural (T1-weighted) scans were first acquired for all participants. Functional (T2-weighted) images for single-band and multi-band scans were then acquired using the following common parameters: TE=25 ms, FOV=23.8 × 23.8 cm; acquisition matrix=70 × 70, no gap, phase encoding=PA, voxel dimensions=3.4 × 3.4 × 3.4 mm. Additional parameters that varied between scanning protocols included: (1) multi-band factor=1, TR=2000 msec, flip angle=77°, number of slices=41; (2) multi-band factor=4, TR=500 msec, flip angle=42°, number of slices=32; (3) multi-band factor=8, TR=500 msec, flip angle=42°, number of slices=41. All FMRI data were reconstructed using 1D-GRAPPA (Blaimer et al., 2013).

#### 2.2.4. Data preprocessing

Data were analyzed with AFNI software (Cox, 1996). After removal of initial calibration volumes (12 s, i.e., 6 vol for MB1 and 24 vol for MB4 and MB8 scans) from each FMRI scan, the following pre-processing steps were performed: (1) slice-timing correction using sync interpolation; (2) motion correction; (3) spatial smoothing (with a 4 mm full-width at half maximum Gaussian kernel; Sacchet and Knutson, 2013); (4) conversion of each voxel’s time series to percent signal change over each run; (5) application of a high-pass filter (removing frequencies below 1 cycle / 90 s or 0.011 Hz). In a supplementary analysis comparing potential effects of temporal smoothing, we applied a band-pass filter with the same high-pass parameters, but varying low-pass parameters (0.25, 0.20, or 0.15 Hz). Affine transformation matrices were then estimated to align functional data to anatomical scans in native space and anatomical scans in individual space to standard group space. These transformations were concatenated and used to transform functional data into a standard group space (i.e., Montreal Neurological Institute or MNI coordinates). Functional data were subsequently visualized in standard space to ensure adequate co-registration (using the MNI anatomical template “mni_icbm152_t1_tal_nlin_asym_09a”).

#### 2.2.5. Data analysis

All functional data were extracted and plotted from five predicted Volumes Of Interest (VOIs): bilateral Nucleus Accumbens (NAcc; CIT168 subcortical atlas; Pauli et al., 2018), bilateral Anterior Insula (AIns; Brainnetome atlas labels 167 & 168; Fan et al., 2016), bilateral Medial PreFrontal Cortex (MPFC; 8-mm diameter spheres centered on MNI: ±5, 50, −1; Knutson et al., 2003), bilateral Primary Visual Cortex (V1; HCP MMP 1.0; Glasser et al., 2016), and left Primary Motor Cortex (M1; AFNI’s TT_Daemon left Precentral Gyrus, transformed to MNI space). Activity from these VOIs was spatially averaged within subject for each task condition, averaged across subjects, and plotted with the standard error for each group for large gain (+$5.00) and loss (−$5.00) trials as well as for non-incentive trials (+$0.00, −$0.00). Trials were separately averaged as a function of whether subjects “hit” or “missed” the target. Consistent with preregistered criteria, brain volumes with excessive motion were excluded (i.e., > 1 mm displacement from one volume acquisition to the next; determined by derivative measures generated by the motion correction algorithm). Values exceeding three standard deviations were also excluded from further analyses. All contrasts from the anticipation period were derived by averaging across time points corresponding to a 4 s anticipation phase (i.e., cue and anticipatory fixation period lasting seconds 0–4 after trial onset) after accounting for a 6 s hemodynamic lag. Contrasts from the outcome period were derived similarly, but averaged time points corresponding to the 2 s outcome phase (i.e., lasting seconds 6–8 after trial onset).

Each subject’s whole brain preprocessed functional data were submitted to a generalized linear regression model including the following four orthogonal regressors of interest: (1) gain (+$5.00) vs. nongain (+$0.00) anticipation (including both cue and anticipatory fixation period); (2) gain (+$5.00) vs. nongain (+$0.00) outcome; (3) loss (−$5.00) vs. nonloss (−$0.00) anticipation; and (4) nonloss (−$0.00) vs. loss (−$5.00) outcome (Knutson et al., 2003). This regression model also included two unit regressors highlighting anticipation and outcome phases of each trial, six motion parameters to control for motion effects, and average activity from cerebrospinal fluid and white matter VOIs to control for physiological noise (Chang and Glover, 2009). Model regressors were convolved with a single gamma hemodynamic response function prior to inclusion in the regression model (Cohen, 1997). To account for fast sampling rates in the multi-band data, we additionally implemented the same temporal autocorrelation algorithm across all acquisition protocols (i.e., generalized least square time series fit with restricted maximum likelihood estimation of the temporal auto-correlation structure using AFNI’s 3dREMLfit). Multiple regression models first fitted each subject’s data in their native space. Maps of resulting *t*-statistics were then converted into Z-scores and warped into standard (MNI) space prior to group comparisons. Within-subject group analyses were subsequently conducted using a mixed effects model with multi-band factor as a fixed effect and subjects as random effects (with AFNI’s 3dANOVA2).

To obtain whole-brain measures of signal and noise in the minimally preprocessed (including slice-timing correction, motion correction, and slight spatial smoothing) unmodeled data, we calculated the average, standard deviation, and Temporal Signal-to-Noise Ratio (TSNR) of the timeseries over the first 100 vol of each functional scan (Chen and Glover, 2015; Demetriou et al., 2018). An additional TSNR calculation involved downsampling all volumes from the multi-band scans acquired every half second (by averaging every four volumes) to the same temporal resolution as single-band scans acquired every two seconds (Todd et al., 2017). Prior to group-level analysis, preprocessed data were warped into standard (MNI) space. As with task-based analyses, whole-brain within-subject group comparisons were implemented using a mixed effects model with multi-band factor as a fixed effect and subjects as random effects.

## 3. Results

### 3.1. Meta-analysis of historical data

Meta-analyses targeted MID task contrasts of interest in predicted VOIs (i.e., gain versus nongain anticipation in the NAcc and gain versus nongain outcome in the MPFC) (Knutson and Greer, 2008) for studies published from 2010 to 2020 (*n* = 44 studies; cumulative *n* = 5005 subjects; Table 2). Standard error values were calculated by dividing the standard deviation (i.e., 1 for standardized scores) by the square root of the sample size.

**Fig. 1.**
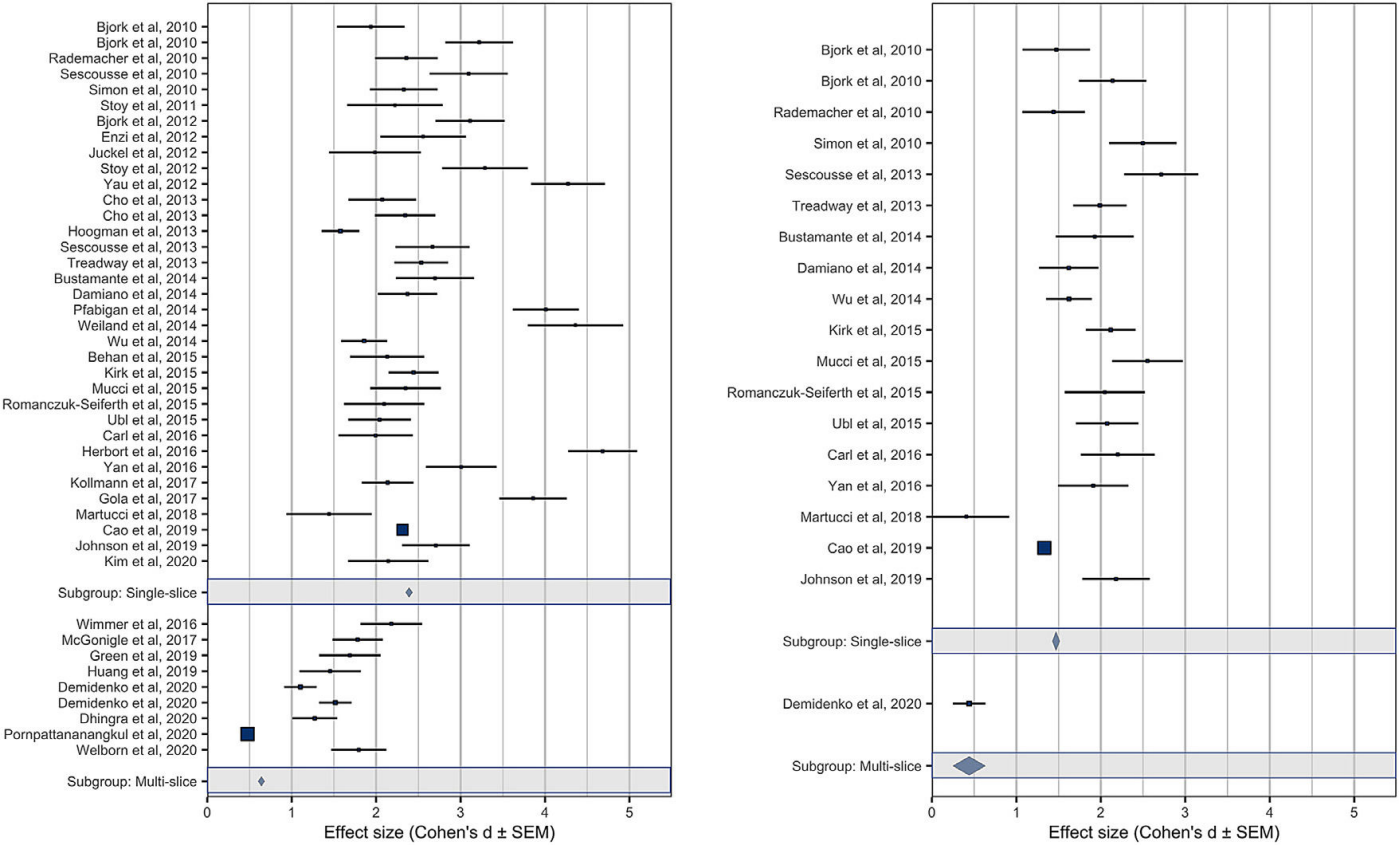
Meta-analytic comparison of NAcc gain versus nongain anticipation contrast peaks (left) and MPFC gain versus nongain outcome contrast peaks (right) across studies. Panels depict single-band (top) versus multi-band (bottom) scans. Single-band studies are ordered by date, and multi-band studies are ordered by MB factor. Gray bars indicate weighted averages (±SEM) for single-band versus multi-band studies. Point sizes indicate sample size (so some studies may appear as squares due to a combination of large samples and small errors).

In direct comparisons, forest plots indicated that although both single-band and multi-band effect sizes robustly exceeded a null effect (i.e., *d* = 0), their magnitudes also differed (Fig. 1). Specifically, gain versus nongain anticipation contrasts in the NAcc all showed robust effect sizes, ranging from “large” to “huge ” (Cohen, 1992), but peak NAcc responses for gain versus nongain anticipation contrasts in single-band studies (weighted mean *d* = 2.39) were significantly larger than those in multi-band studies (weighted mean *d* = 0.64; *t*(42)=4.16, *p*<0.001). Similarly, peak MPFC responses for gain versus nongain outcomes in single-band studies (weighted mean *d* = 1.47) were larger than in the multi-band study (single study *d* = 0.44). Thus, single-band effect sizes were at least three times as large as multi-band effect sizes.

**Fig. 2.**
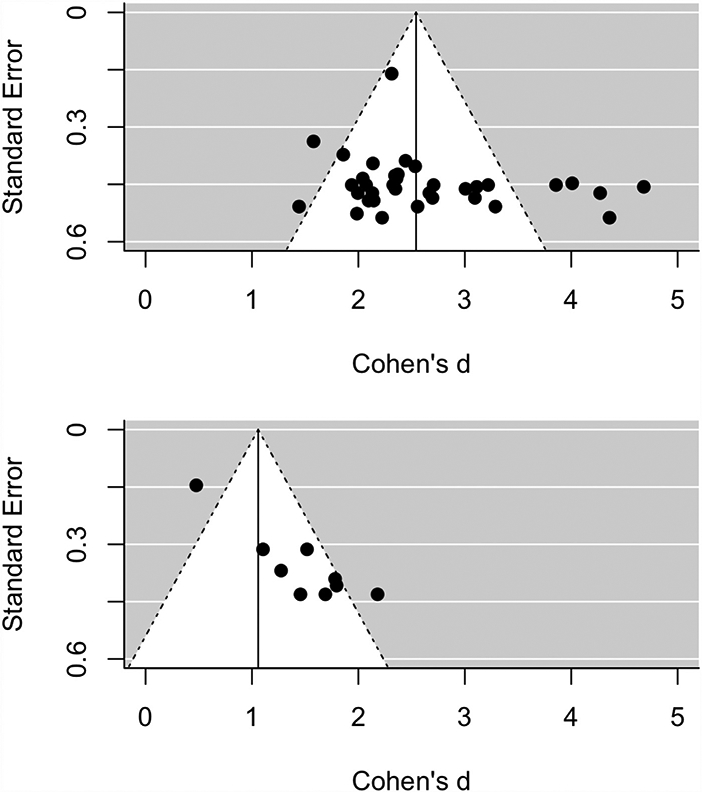
Funnel plots of study effect size versus standard error for the NAcc gain versus nongain anticipation contrast. Panels depict single-band (top) and multi-band (bottom) studies.

Next, we examined whether these differences might be attributable to a publication bias favoring significant effects in single-band studies. Funnel plots indicated that for the peak NAcc gain versus nongain anticipation contrasts, single-band studies showed less (rather than more) evidence of publication bias than did multi-band studies (i.e., fewer studies to the right of the funnel; Fig. 2). Quantitative comparison of the association of effect size with standard error (Egger et al., 1997) revealed that although single-band studies showed some evidence of association (*z* = 2.64, *p* = 0.0083), multi-band studies showed even stronger evidence (*z* = 5.64, *p*<0.0001), and that the difference between multi-band and single-band associations was significant (*z* = 3.00, *p*<0.01). Since these tests suggested that publication bias was larger (rather than smaller) for multi-band studies, selection effects could not account for the smaller effect sizes observed in multi-band studies.

Multivariate statistical tests further confirmed the robustness of these findings against potential confounds. Specifically, peak NAcc responses to gain versus nongain anticipation did not significantly differ as a function of scanner brand (Siemens *d* = 2.38±0.18 versus GE *d* = 2.45±0.24 versus Philips *d* = 2.34±0.45, *F*(2,41)=2.15, *p* = 0.13) or field strength (1.5 T *d* = 2.46±0.16 versus 3.0 T *d* = 2.38±0.17, *t*(42)=0.25, *p* = 0.80). Statistical comparisons of the localization of peak coordinates further indicated that activation foci did not shift as a function of slice acquisition protocol (e.g., Sacchet and Knutson 2013). Specifically, for the gain versus nongain contrast in the ventral striatum, peak coordinates for single-band versus multi-band studies did not differ in x (*t*(42)=0.67, *p* = 0. 51), y (*t*(42)=−0.22, *p* = 0. 83), or z (*t*(42)=−0.73, *p* = 0.47) coordinates.

Statistical comparisons did, however, reveal some predicted but distinct effects. For instance, adolescent versus adult samples trended towards showing reduced NAcc gain versus nongain anticipation contrast peaks (adolescent *d* = 1.90±0.41 versus adult *d* = 2.51±0.13, *t*(42)=1.80; *p* = 0.08), consistent with previous developmental comparisons using the MID task (e.g., Bjork et al. 2004). Separate regression analyses also confirmed that increases in publication date (standardized (std.) *r*=−0.42; *t*(42)=−2.98, *p* = 0.005) and number of subjects (std. *r*=−0.31; *t*(42)=−2.14, *p* = 0.038) were associated with decreased effect sizes, but that image acquisition flip angle (std. *r* = 0.36; *t*(42)=2.50, *p* = 0. 016) was associated with increased effect sizes. Voxel size, however, was not significantly associated with effect sizes as expected (std. *r*=−0.03; *t*(42)=−1.16, *p* = 0.252). Next, potentially correlated confounds (i.e., subject age, publication date, sample size, and flip angle) were included in a multivariate analysis to verify the robustness of the association of single-band versus multi-band scanning with effect size. An ANalysis of COVAriance (ANCOVA) which included all of these correlated variables revealed that only variation in single-band versus multi-band scanning continued to be associated with NAcc response during gain versus nongain anticipation (*F*(1,37)=7.68, *p* = 0.009; Table 3). Within multi-band studies (*n* = 9), the association of multi-band factor with NAcc gain versus nongain effect size was negative as predicted, but not significant (r(9) =−0.40, *p* = 0.28), possibly due to the small number of multi-band studies available for analysis (Todd et al., 2017). An insufficient number of available studies including the gain versus nongain outcome contrast precluded parallel meta-analysis of gain outcome findings.

Together, meta-analytic findings implied that use of multi-band versus single-band scans can compromise detection of brain responses to reward anticipation and possibly outcomes. The substantial decrement (of over half) in effect sizes was not attributable to potential confounds (e.g., scanner brand or field strength) or other correlated variables (e.g., subject age, publication date, sample size, flip angle).

### 3.2. Direct comparison of original data

**Fig. 3.**
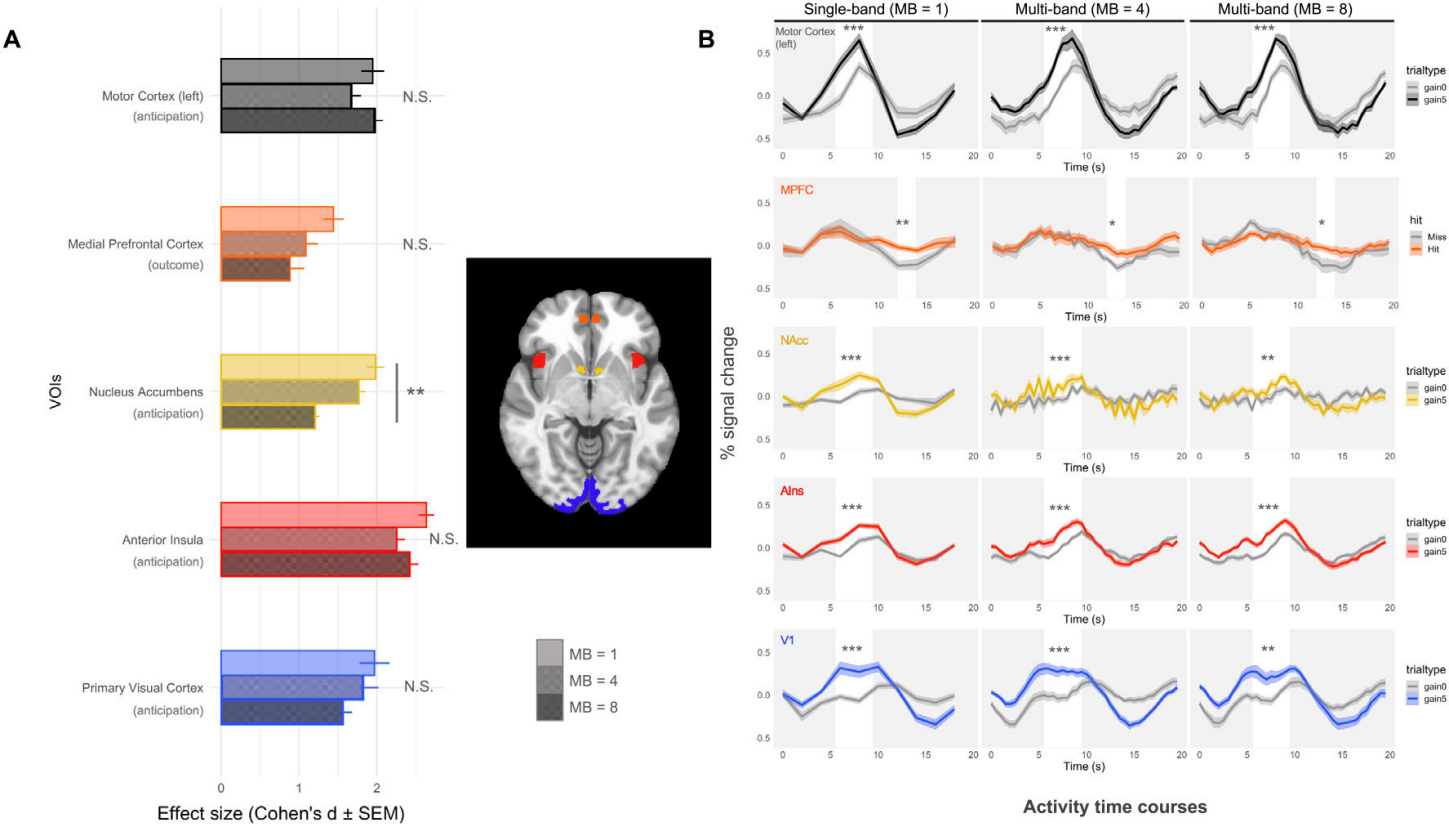
Effects and activity time courses for large gain (+$5.00) versus nongain (+$0.00) contrasts across VOIs. (A) Effect size estimates (Cohen’s *d* ± standard error) are calculated separately for large gain versus nongain anticipation or outcome (for MPFC) contrasts in each VOI. Significant differences are marked with * (* *p*<0.05; **p<0.01; ***p<0.001); (B) VOI activity time course data for single-band (MB1) versus multi-band (MB4 and MB8) data (*n* = 12; within-subjects). In all plots, time on the x-axis is seconds after trial onset (at second 0). White bars highlight time points of interest corresponding to either anticipation or outcome periods (after a 6 s lag for the hemodynamic delay).

**Fig. 4.**
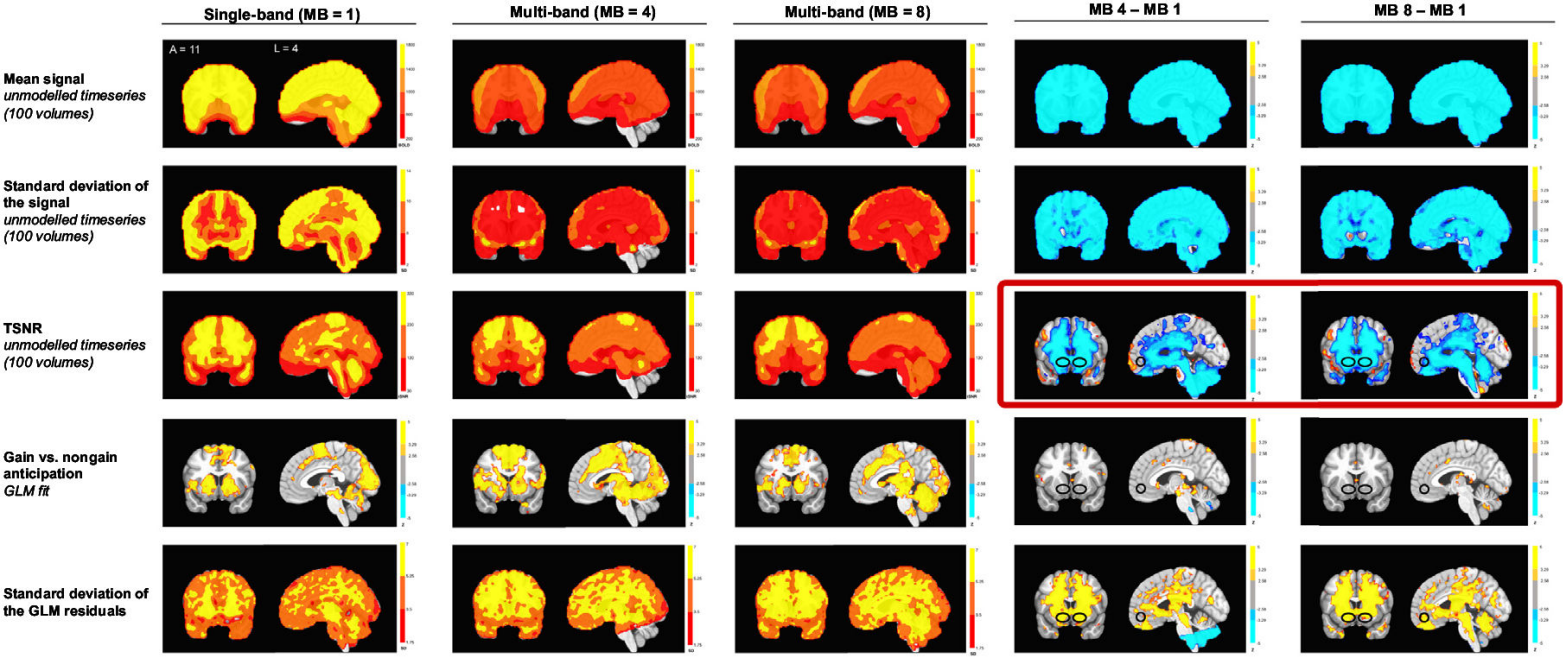
Whole brain signal and noise measures for baseline and modeled data from FMRI scans during the MID task. Rows depict: (1) mean overall activity; (2) standard deviation of overall activity; (3) temporal signal-to-noise ratio; (4) gain versus nongain anticipation coefficient (thresholded at *p* < 0.01 with a cluster size of 4); and (5) standard deviation of the gain versus nongain anticipation coefficient.

Although the meta-analysis indicated that multi-band scanning might compromise detection of reward-related activity, reasons for this compromise remained unclear. For instance, the findings did not clarify whether the problem resulted from decreased signal or increased noise and could not elucidate whether the problem was localized in space (e.g., influencing activity in the center of the brain more than the periphery) or time (e.g., occurring throughout the scan or only during particular task conditions). Thus, we sought to reproduce and diagnose the problem by directly comparing original MID task data acquired with single-band and multi-band scanning protocols. To test the critical hypotheses, activity time course data were extracted from predicted VOIs, as well as from sensory and motor (i.e., V1 and left M1) control VOIs (see Fig. 3). Whole-brain analyses further contrasted statistical estimates of both task-independent and task-dependent signal and noise (see Fig. 4).

As in meta-analytic findings, group contrasts revealed large or huge effect sizes for gain versus nongain anticipation contrasts in the NAcc VOI for single-band data, but significantly reduced effect sizes for multi-band scans (single-band: *d* = 1.98, *SEM*=0.11; MB4: *d* = 1.77, *SEM*=0.08; MB8: *d* = 1.21, *SEM*=0.05; *std*. *ß*=−3.00, *p* = 0.004). The effect size for gain versus nongain outcome contrasts in the MPFC VOI was also slightly higher for single-band than for multi-band scans, but this difference was not significant (single-band: *d* = 1.44, *SEM*=0.13, MB4: *d* = 1.09, *SEM*=0.15; MB8: *d* = 0.88, *SEM*=0.18; *std*. *ß*=−0.37, *p* = 0.70). Previous research using the MID task has also documented significant AIns, V1, and M1 responses during gain versus nongain anticipation (e.g., Knutson et al. 2003). These control VOIs yielded large effect sizes which did not significantly differ between single-band and multi-band scans (Fig. 3; all *ds*> 1.5, all *std*. *ß* values<1.82, all *p*-values >0.05). Statistical tests also controlled for within-subject order effects of acquisition protocols and revealed no significant effects of order on brain activity, except for the AIns VOI (*std*. *ß*=2.44, *p* = 0.016, all *std*. *ß* values for other VOIs <1.26, all other *p*-values >0.20). Together, these findings suggest spatial specificity, since multi-band versus single-band scans compromised detection of reward-related activity in mesolimbic regions of interest (e.g., near the NAcc), but not in regions closer to the edge of the brain (e.g., V1 and M1; which lie closer to the radiofrequency coils).

While visual inspection of the single-band activity time courses revealed the predicted increases in activity in the NAcc and AIns (as well as in primary visual and motor cortices) during gain versus nongain anticipation, and also in MPFC activity in response to gain versus nongain outcomes (e.g., Wu et al. 2014), high frequency oscillatory activity in the mesolimbic VOIs was also apparent (Fig. 3B). Inspection of periodograms of these regions’ activity confirmed a frequency peak around 1 Hz, (Supplemental Fig. S1). To explore whether temporal filtering of these frequencies could reduce NAcc noise in multi-band scans, we applied low-pass temporal filters (of 0.25, 0.20, and 0.15 Hz) to the data (Fig. S2). Subsequent analyses revealed, however, that low-pass filtering had no significant effect on findings in any region (all *std*. *ßs* < 0.33, all *p*-values > 0.70). Instead, scan sequence continued to exert a main effect on NAcc (*std. ß*=−3.07, *p* = 0.002) as well as V1 (*std. ß*=−;2.66, *p* = 0.008) activity (Fig. S3).

To diagnose the spatial and temporal specificity of the findings, signal and noise indices were compared in both unmodeled and modeled data. First, the mean, standard deviation, and Temporal Signal-to-Noise Ratio (TSNR) of the unmodeled activity timeseries in each of the functional scans were calculated. Next, coefficients for the gain versus nongain anticipation contrast and the standard deviation of the regression residuals were calculated (Fig. 4). While both the mean and standard deviation of the raw signal were lower across the whole brain in the multi-band data, TSNR (calculated as the ratio of the mean over standard deviation over the first 100 vol (Demetriou et al., 2018; Fig. 4) or per unit time (in two second increments; Todd et al., 2016; Fig. S4) was specifically lower in subcortical regions that overlapped with the mesolimbic VOIs. In the modeled multi-band scan data, gain versus nongain anticipation contrast coefficients were not significantly lower in the NAcc but the standard deviation of task fit residuals was larger, particularly in mesolimbic regions. As with activity time course plots, these analyses suggested that model-independent noise in mesolimbic regions might compromise detection of reward-related FMRI activity in multi-band scans.

**Fig. 5.**
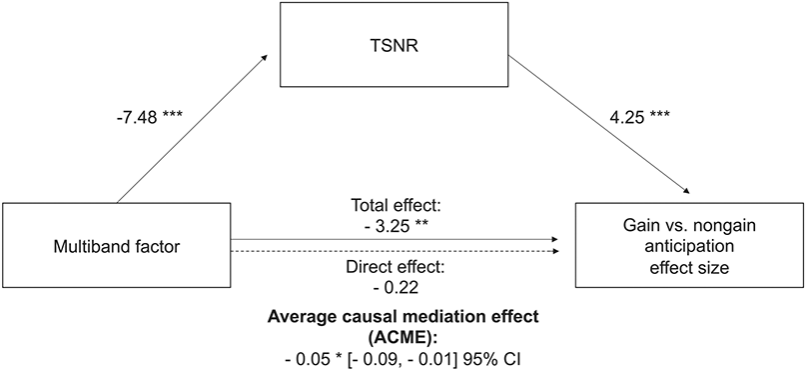
Temporal signal-to-noise ratio mediates the influence of multi-band scanning on reward-related activity in the Nucleus Accumbens. * *p* < .05; ** *p* < .01; *** *p* < *.001*

Finally, a mediation analysis tested whether overall TSNR might statistically mediate the influence of multi-band acquisition on reward-related activity in the NAcc. A linear mixed effects regression on NAcc gain versus nongain activity included spatially averaged TSNR in the NAcc VOI as a mediator, with multi-band factor and order as fixed effects and subjects as random intercepts. Multi-band factor was associated with decreased effect size for NAcc gain versus nongain anticipation (*std.ß*=−3.25, *p*<0.007) as well as with decreased NAcc TSNR (*std. ß*=−7.48, *p*<0.00001). Further, NAcc TSNR was positively associated with NAcc gain versus nongain anticipation effect size (*std. ß*=4.25, *p*<0.001). After statistically controlling for TSNR, the association between multi-band factor and NAcc gain versus nongain anticipation effect size was significantly diminished (*std. ß*=−3.25, *p*<0.002) and rendered nonsignificant. A bootstrapped causal mediation model with 3000 simulations (R package *‘mediation 4.5.0′*; Tingley et al., 2014) revealed that NAcc TSNR could fully mediate the association between multi-band factor and NAcc gain versus nongain anticipation effect size (average causal mediation effect: *ß*=−0.046, *p* = 0.017, 95% quasi-Bayesian Confidence Interval [−0.09, −0.01]), with the mediator accounting for 88.54% of the total effect (Fig. 5). Similar mediation results held using a different calculation of TSNR per unit time (Fig. S5).

## 4. Discussion

In a meta-analysis of historical data as well as in a preregistered analysis of original data, we compared the influence of single-band versus multi-band FMRI scanning protocols on the detectability of reward-related brain activity. Meta-analytic results indicated that relative to single-band scans, multi-band scans compromised detection of mesolimbic activity during reward anticipation. This compromise was not associated with other potential confounds (e.g., scanner brand or field strength), and could not be attributed to other covarying factors (e.g., subject age, publication date, sample size, or flip angle). While this compromise may worsen as a function of the strength of multi-band factor within multi-band protocols (e.g., Demetriou et al. 2018 and Todd et al. 2017), not enough studies were available to directly test this association.

Direct comparison of single- versus multi-band FMRI datasets acquired in the same subjects during the same session on the same scanner provided further evidence that multi-band scanning compromised detection of reward-related mesolimbic activity. This compromise appeared most clearly in the center of the brain and was mediated by increased temporal noise throughout the scan, consistent with earlier research (Risk et al., 2018; Todd et al., 2017). Prior investigators have attributed multi-band induced signal compromise to two sources: (1) decreased longitudinal signal associated with faster volume acquisitions and echo times; and (2) increased high frequency thermal noise amplified by geometric factors in regions with overlapping stimulation (Todd et al., 2017). In the current research, signal compromise could not be attributed to decreased longitudinal magnetization, since the time to acquire images and echo time did not differ between the two multi-band factor scan protocols (Table 1), yet the higher multi-band factor scan showed more compromise than the lower. Instead, the subcortical localization of baseline noise (Fig. 4) and mediation of multi-band induced NAcc signal compromise by TSNR throughout the scan (Fig. 5) point to geometric factor induced noise as the most likely culprit. Although temporal filtering smoothed the appearance of activity time courses, it could not rescue decrements in detectability associated with multi-band scanning (Risk et al., 2021).

While previous studies have examined the impact of multi-band scans on FMRI data in humans at rest (Risk et al., 2018, 2021) and during sensorimotor and cognitive tasks (Demetriou et al., 2018; Todd et al., 2017), this study specifically focused on the influence of multi-band scanning on FMRI responses during a reward task (Knutson et al., 2001). Just as FMRI vision processing localizer tasks can reliably recruit primary visual cortical responses, reward processing localizer tasks can consistently recruit mesolimbic responses (i.e., the NAcc and MPFC) (Bartra et al., 2013; Clithero and Rangel, 2014; Knutson and Greer, 2008). The MID task selectively and reliably activates these mesolimbic brain regions during anticipation and receipt of monetary rewards (Wu et al., 2014), and this activity has demonstrated validity with respect to predicting healthy decision-making (Knutson and Stallen, 2018), as well as psychiatric symptoms (Knutson and Heinz, 2015). Together, these findings point to a literal hole in recent multi-band neuroimaging research, a hole which lies at the center of the brain. Thus, the findings have implications for investigators who seek to study value-based choice in the context of healthy decision-making, as well as for those who wish to measure neural markers of individual differences in affect and motivation in the context of psychiatric symptoms.

Strengths of this research include elicitation of robust neural responses to reward probes, control for potential confounds (e.g., scanner brand, field strength, task, time of measurement, between-subject variability), preregistration of predictions and power, and convergence of findings across meta-analysis of historical findings as well as analysis of original data. Potential limitations include too few studies in the meta-analysis to statistically compare the influence of different multi-band scan protocols on detection of responses to gain versus nongain outcomes in the frontal cortex or to compare the influence of increasing multi-band factors (although earlier work has documented multiband-induced reductions in task-related signal in the MPFC; Risk et al., 2018). While the direct comparison of original data required a relatively small sample size (*n* = 12), meta-analysis supported the sample’s adequacy, consistent with large effect sizes typically observed in the NAcc for the gain versus nongain anticipation contrast (e.g., weighted effect size average of 2.4 for single-band data) as well as with the preregistered power analyses. Although this research specifically focused on the MID task, given its robust elicitation of mesolimbic activity, findings should generalize to other tasks that reliably recruit mesolimbic activity. The mediating role of baseline temporal signal-to-noise ratio suggests that compromised detection of mesolimbic activity should not be limited to task-related FMRI (Demetriou et al., 2018; Todd et al., 2017), and may also extend to activity during rest (Risk et al., 2018). Indeed, a comprehensive recent comparison revealed artifacts in the center of the brain at rest, leading the investigators to caution that multi-band scanning protocols might obscure subcortical activity (Risk et al., 2021).

With respect to clinical applications, researchers have sought to use FMRI activity in mesolimbic regions (associated with both tasks and rest) to index individual differences. The validity of these measures as indices of experience or behavior is bounded by their reliability (Knutson and Heinz, 2015). Studies of development and psychiatric symptoms have used tasks designed to probe mesolimbic responses to index individual differences, and some research using these tasks has acquired data with multi-band scanning protocols. For example, several large longitudinal studies have adopted multi-band scanning protocols (e.g., the Human Connectome Project (HCP; Van Essen et al., 2013), the Adolescent Brain Cognitive Development (ABCD) Study (Casey et al., 2018), and the Dunedin Study (Caspi et al., 2020)), which may compromise researchers’ ability to reliably measure and track individual differences in mesolimbic activity. Further, switching from single-band to multi-band scanning during a study with repeated measures might also compromise reliability (Elliott et al., 2020).

If multi-band scans compromise FMRI assessment of mesolimbic activity, how can researchers address this issue? Those who have not begun to collect data might initially opt for single-band over multi-band scanning protocols (or minimally, multi-band protocols with low multi-band factors). But if data have already been collected, researchers might at least diagnose problems by probing mesolimbic reward responses with a reliable task and then visualizing activity time courses to check for high frequency noise. Raw activity from mesolimbic regions could also be probed for evidence of high frequency oscillatory activity (e.g., with periodograms) or low temporal signal-to-noise ratio. The benefits of ensuring adequate temporal signal to noise ratio could be substantial, since the current findings suggest that opting for single-band instead of multi-band scans might increase statistical power, which could substantially reduce the number of subjects or scanning time required to achieve statistical significance. On the one hand, these findings offer good news by reinforcing the power, stability, and robustness of earlier FMRI findings related to reward processing. On the other hand, these results suggest caution in interpreting more recent findings acquired with rapid acquisition methods, and imply that the benefits of increased speed might come at a cost of lost signal.

## Supplementary Material

1

## Figures and Tables

**Table 1:** Scanning parameters used for each sequence. MB = Multi-Band factor; FOV = Field Of View; TE = Time to Echo; TR = Time to Repetition.

	MB 1	MB 4	MB 8
Voxel size	3.4 × 3.4 × 3.4	3.4 × 3.4 × 3.4	3.4 × 3.4 × 3.4
Freq. FOV	23.8	23.8	23.8
Phase FOV	1	1	1
TE	25ms	25ms	25ms
Phase encoding	PA	PA	PA
Base resolution	70 × 70 at 3.4mm	70 × 70 at 3.4mm	70 × 70 at 3.4mm
Pixel bandwidth	7143Hz	7143Hz	7143Hz
Echo spacing	0.5	0.5	0.5
Flip angle	77	42	42
Number of slices	41	32	41
TR	2000ms	500ms	500ms

**Table 2:** FMRI studies of reward processing included in the meta-analysis (ranging from 2010 to 2020). FWHM = Full Width at Half Maximum; Gvnant = Gain versus nongain anticipation contrast; Gvnout = Gain versus nongain outcome contrast; SE = Standard Error; VOI = Volume of Interest.

Study	Year	Subjects	N	Scanner brand	Field strength	Band	Band factor	FWHM	Voxel dimensions	Voxel size	Flip angle	Gvnant effect size	Gvnant SE	Gvnant VOI	Gvnout effect size	Gvnout SE	Gvnout VOI
Behan et al.	2015	Adults	20	Philips	3	single-band	1	4.2	3.5 × 3.5 × 3.5	42.875	90	2.131272168	0.223606798	NAcc			
Bjork et al.	2010	Adolescents	24	GE	3	single-band	1	8	3.75 × 3.75 × 3.75	52.73438	90	1.937515935	0.204124145	NAcc	1.472528792	0.204124145	MPFC
Bjork et al.	2010	Adults	24	GE	3	single-band	1	8	3.75 × 3.75 × 3.75	52.73438	90	3.219045524	0.204124145	Caudate	2.139357888	0.204124145	MPFC
Bjork et al.	2012	Adults	23	GE	3	single-band	1	8	3.75 × 3.75 × 3.75	52.73438	90	3.111451254	0.208514414	NAcc			
Bustamante et al.	2014	Adults	18	Siemens	1.5	Single-band	1	8	3 ×3 ×3	27	90	2.696433859	0.23570226	VS	1.92804449	0.23570226	MPFC
Cao et al.	2019	Adolescents	1510	Siemens	3	single-band	1	5	3.44 × 3.44 × 3.4	40.23	75	2.312731009	0.025734251	NAcc	1.329871816	0.025734251	VMPFC
Carl et al.	2016	Adults	20	GE	3	single-band	1	5	3.75 × 3.75 × 4.0	56.25	60	1.994572636	0.223606798	NAcc	2.20029089	0.223606798	ACC
Cho et al.	2013	Adolescents	24	GE	3	single-band	1	8	3.75 × 3.75 × 4	56.25	90	2.072633276	0.204124145	NAcc			
Cho et al.	2013	Adults	30	GE	3	single-band	1	8	3.75 × 3.75 × 4.0	56.25	90	2.343475168	0.182574186	NAcc			
Damiano et al.	2014	Adults	31	GE	3	Single-band	1	5	4 × 4 × 4	64	77	2.370789987	0.179605302	NAcc	1.620039824	0.179605302	ACC
Enzi et al.	2012	Adults	15	Siemens	1.5	single-band	1	8	3.8 × 3.8 × 3.8	54.872	90	2.556169008	0.25819889	VS			
Gola et al.	2017	Adults	24	Siemens	3	single-band	1	8	3.5 × 3.5 × 3.5	42.875	90	3.857142857	0.204124145	NAcc			
Herbort et al.	2016	Adults	23	Siemens	3	Single-band	1	8	3 × 3 × 3	27	70	4.681887731	0.208514414	NAcc			
Hoogman et al.	2013	Adults	77	Siemens	1.5	Single-band	1	8	3.5 × 3.5 × 3	36.75	90	1.578380249	0.113960576	VS			
Johnson et al.	2019	Adults	24	GE	1.5	Single-band	1	4	3.44 × 3.44 × 4.0	47.33	90	2.706686166	0.204124145	NAcc	2.180045871	0.204124145	MPFC
Juckel et al.	2012	Adults	13	Siemens	1.5	single-band	1	8	4 × 4 × 3.3	52.8	90	1.986084926	0.277350098	VS			
Kim et al.	2020	Adults	17	Siemens	3	single-band	1	6	3.1 × 3.1 × 3	28.83	90	2.144014925	0.242535625	NAcc			
Kirk et al.	2015	Adults	44	Siemens	3	single-band	1	8	3.4 × 3.4 × 4	46.24	90	2.443027097	0.150755672	Caudate	2.116680156	0.150755672	VMPFC
Kollmann et al.	2017	Adults	41	Siemens	3	Single-band	1	7	2.3 × 2.3 × 3	15.87	90	2.136457063	0.156173762	VS			
Martucci et al.	2018	Adults	15	GE	3	Single-band	1	4	3.4 × 3.4 × 4	46.24	76	1.442831815	0.25819889	VS	0.407883101	0.25819889	MPFC
Mucci et al.	2015	Adults	22	Philips	3	single-band	1	6	3.59 × 3.59 × 4	51.55	90	2.348024499	0.213200716	VS	2.553149316	0.213200716	MPFC
Pfabigan et al.	2014	Adults	25	Siemens	3	Single-band	1	8	1.5 × 1.5 × 3	6.75	60	4.008998212	0.2	VS			
Rademacher et al.	2010	Adults	28	Philips	1.5	Single-band	1	6	3.75 × 3.75 × 3.8	53.4375	90	2.358498312	0.188982237	NAcc	1.440044642	0.188982237	ACC
Romanczuk-Seiferth et al.	2015	Adults	17	Siemens	3	Single-band	1	8	3.5 × 3.5 × 3.0	36.75	80	2.0955078	0.242535625	Putamen	2.047000675	0.242535625	ACC
Sescousse et al.	2010	Adults	18	Siemens	1.5	Single-band	1	10	3.4 × 3.4 × 4	46.24	90	3.094754575	0.23570226	VS			
Sescousse et al.	2013	Adults	20	Siemens	1.5	Single-band	1	10	3.4 × 3.4 × 4	46.24	90	2.666666667	0.223606798	VS	2.716282289	0.223606798	Ant.Orb.Gyrus
Simon et al.	2010	Adults	24	Siemens	3	single-band	1	8	3 × 3 × 3	27	80	2.327020861	0.204124145	VS	2.49800268	0.204124145	MPFC
Stoy et al.	2011	Adults	12	Siemens	1.5	single-band	1	8	4 × 4 × 3.3	52.8	90	2.222798536	0.288675135	Putamen			
Stoy et al.	2012	Adults	15	Siemens	1.5	single-band	1	8	4 × 4 × 3.3	52.8	90	3.287313276	0.25819889	Putamen			
Treadway et al.	2013	Adults	38	Philips	3	Single-band	1	6	1.87 × 1.87 × 2.75	9.62	90	2.533898598	0.162221421	VS	1.988834623	0.162221421	MPFC
Ubl et al.	2015	Adults	28	Siemens	3	Single-band	1	6	2.3 × 2.3 × 3	15.87	90	2.041008154	0.188982237	Caudate	2.075024957	0.188982237	ACC
Weiland et al.	2014	Adolescents	12	GE	3	Single-band	1	6	3.12 × 3.12 × 4	38.94	90	4.359854043	0.288675135	VS			
Wu et al.	2014	Adults	52	GE	1.5	single-band	1	4	3.75 × 3.75 × 3.75	52.73438	90	1.858245657	0.138675049	NAcc	1.622498074	0.138675049	MPFC
Yan et al.	2016	Adults	22	Siemens	3	single-band	1	8	3.4 × 3.4 × 4	46.24	90	3.006130101	0.213200716	VS	1.910278419	0.213200716	MPFC
Yau et al.	2012	Adults	20	GE	3	single-band	1	6	3.12 × 3.12 × 3.12	30.37133	90	4.271720965	0.223606798	VS			
Wimmer et al.	2016	Adults	29	Siemens	3	Multi-band	2	6	2 × 2 × 2	8	60	2.18006327	0.185695338	VS			
McGonigle et al.	2017	Adults	43	Siemens	3	Multi-band	2	6	3.516 × 3.516 × 3.516	43.46569	80	1.781183301	0.15249857	NAcc			
Dhingra et al.	2020	Adults	54	Siemens	3	Multi-band	3	8	3 × 3 × 3	27	62	1.273734666	0.136082763	VS			
Green et al.	2019	Adults	14	Siemens	3	Multi-band	3	4	1.5 × 1.5 × 1.5	3.375	80	1.68982758	0.185695338	Caudate			
Demidenko et al.	2020	Adolescents	104	GE	3	Multi-band	6	5	2.4 × 2.4 × 2.4	13.824	52	1.103568792	0.098058068	NAcc	0.441261304	0.098058068	MPFC
Demidenko et al.	2021	Adolescents	104	GE	3	Multi-band	6	5	2.4 × 2.4 × 2.4	13.824	52	1.517407088	0.098058068	NAcc			
Huang et al.	2019	Adolescents	29	Siemens	3	Multi-band	6	6	2.4 × 2.4 × 2.4	13.824	77	1.455851451	0.185695338	VS			
Pornpattananangkul et al.	2020	Adolescents	2222	Siemens	3	Multi-band	6	2	2.4 × 2.4 × 2.4	13.824	52	0.476595745	0.021214264	NAcc			
Welborn et al.	2020	Adults	36	Siemens	3	Multi-band	8	6	2 × 2 × 2	8	52	1.795107637	0.166666667	NAcc			

**Table 3:** ANCOVA of NAcc gain versus nongain anticipation effect size as a function of single-band versus multi-band scanning protocol and potential confounds.

Cases	Sum of Squares	df	Mean Square	F	p
Age	0.051	1	0.051	0.089	0.767
Band	4.394	1	4.394	7.676	0.009
Age * Band	0.521	1	0.521	0.910	0.346
Date	0.165	1	0.165	0.289	0.594
Sample size	0.903	1	0.903	1.578	0.217
Flip angle	0.487	1	0.487	0.851	0.362
Residuals	21.182	37	0.572		

Note. Type III Sum of Squares.
