# Supplementary material for: Multi-band FMRI compromises detection of mesolimbic reward responses
Source: Neuroimage. Author manuscript; Available in PMC 2021 Dec 1. (PMC8626533; doi:10.1016/j.neuroimage.2021.118617)
Supplement: 1 [file NIHMS1756243-supplement-1.docx]

Table S1. Studies excluded from the meta-analysis.

| **Study** | **Year** | **Reason for exclusion** |
| --- | --- | --- |
| Dillon et al. | 2010 | No t or z-values |
| Alves et al. | 2010 | Only placebo and treatment condition (no true controls) |
| Andrews et al. | 2011 | Contrast not available: only group differences |
| Jia et al. | 2011 | Contrast not available: only group differences |
| Figee et al. | 2011 | No t or z-values |
| Jung et al. | 2011 | Contrast not available: only group differences |
| Balodis et al. | 2012 | No contrasts for striatal regions; see Supplement |
| Filbey et al. | 2013 | Missing values for controls in Table 2 |
| Kaufmann et al. | 2013 | Missing exact values ("< 8"); see Supplement |
| Saji et al. | 2013 | Only placebo and treatment condition (no true controls) |
| Patel et al. | 2013 | Correlated activity; not task-based |
| Edel et al. | 2013 | Contrast not available |
| Boecker et al. | 2014 | Contrast not available |
| Funayama et al. | 2014 | Only placebo and treatment conditions (no true controls) |
| Maresh et al. | 2014 | Contrast not available |
| Trost et al. | 2014 | Conditioned reward stimuli-- therefore required learning |
| Yip et al. | 2014 | Contrast not available |
| Richey et al. | 2014 | Only mean z-value reported (across groups) |
| Mason et al. | 2014 | Contrast not available |
| Behan et al. | 2015 | No contrasts for striatal regions; see Supplement |
| Hägele et al. | 2015 | No contrasts available for controls |
| Hanssen et al. | 2015 | Contrast not available |
| Kappel et al. | 2015 | Contrast not available: only group differences |
| Spaniol et al. | 2015 | Contrast not available |
| Van Hulst et al. | 2015 | Modified MID task |
| Joseph et al. | 2016 | Nonstandard contrasts, no percent signal change |
| Mori et al. | 2016 | Contrast not available: only group differences |
| Berghorst et al. | 2016 | Contrast not available |
| Van Duin et al. | 2016 | No contrasts for striatal regions |
| Dutra et al. | 2017 | Contrast not available |
| Kocsel et al. | 2017 | No striatal regions for gain anticipation; see Table 2 |
| Büchel et al. | 2017 | Subsample of IMAGEN reported in Cao et al. (2017), which was included in meta-analysis |
| Holiga et al. | 2018 | Contrasts or percent signal change not available (only reliability) |
| Held-Poschardt et al. | 2018 | Contrast not available for controls |
| Fede et al. | 2019 | No control subjects |
| Schmidt et al. | 2020 | Contrast not available |
| Doell et al. | 2020 | Contrast not available |
| Lawn et al. | 2020 | Only placebo and treatment condition (no true controls) |
| Wang et al. | 2020 | Only placebo and treatment condition (no true controls) |
| Le et al. | 2020 | Contrast not available |
| Vaidya et al. | 2020 | Modified MID task |
| Grimm et al. | 2020 | Only placebo and treatment condition (no true controls) |
| Kryza-Lacombe et al. | 2020 | Contrast not available |
| Kostandyan et al. | 2020 | Contrast not available |
| Volman et al. | 2020 | Only placebo and treatment condition (no true controls) |
| Boukezzi et al. | 2020 | No true controls |
| Millman et al. | 2020 | Contrast not available |

Table S2. Volume of interest activity in response to gain anticipation and outcomes (n=12).

|  |  | **MB1** | **MB4** | **MB8** |  |
| --- | --- | --- | --- | --- | --- |
| **Motor Cortex (left)** | +$5 gain anticipation | 0.51 (0.22) | 0.49 (0.24) | 0.45 (0.19) |  |
|  | +$0 gain anticipation | 0.15 (0.14) | 0.17 (0.12) | 0.14 (0.11) |  |
|  |  |  |  |  |  |
| **Medial Prefrontal Cortex** | +$5 gain outcome | -0.02 (0.20) | -0.07 (0.14) | -0.03 (0.07) |  |
|  | +$0 gain outcome | -0.24 (0.17) | -0.22 (0.11) | -0.22 (0.26) |  |
|  |  |  |  |  |  |
| **Nucleus Accumbens** | +$5 gain anticipation | 0.19 (0.12) | 0.15 (0.09) | 0.12 (0.07) |  |
|  | +$0 gain anticipation | -0.01 (0.08) | 0.01 (0.06) | 0.02 (0.09) |  |
|  |  |  |  |  |  |
| **Anterior Insula** | +$5 gain anticipation | 0.18 (0.08) | 0.20 (0.09) | 0.19 (0.07) |  |
|  | +$0 gain anticipation | -0.01 (0.06) | 0.04 (0.05) | 0.03 (0.06) |  |
|  |  |  |  |  |  |
| **Primary Visual Cortex** | +$5 gain anticipation | 0.29 (0.22) | 0.28 (0.14) | 0.24 (0.18) |  |
|  | +$0 gain anticipation | -0.04 (0.09) | 0.01 (0.015) | 0.003 (0.11) |  |
|  |  | *Mean (SD)* | | | |

Figure S1. Periodogram of Nucleus Accumbens activity power for each subject. Green rectangles highlight task-related frequencies, whereas red rectangles highlight task-unrelated frequencies.


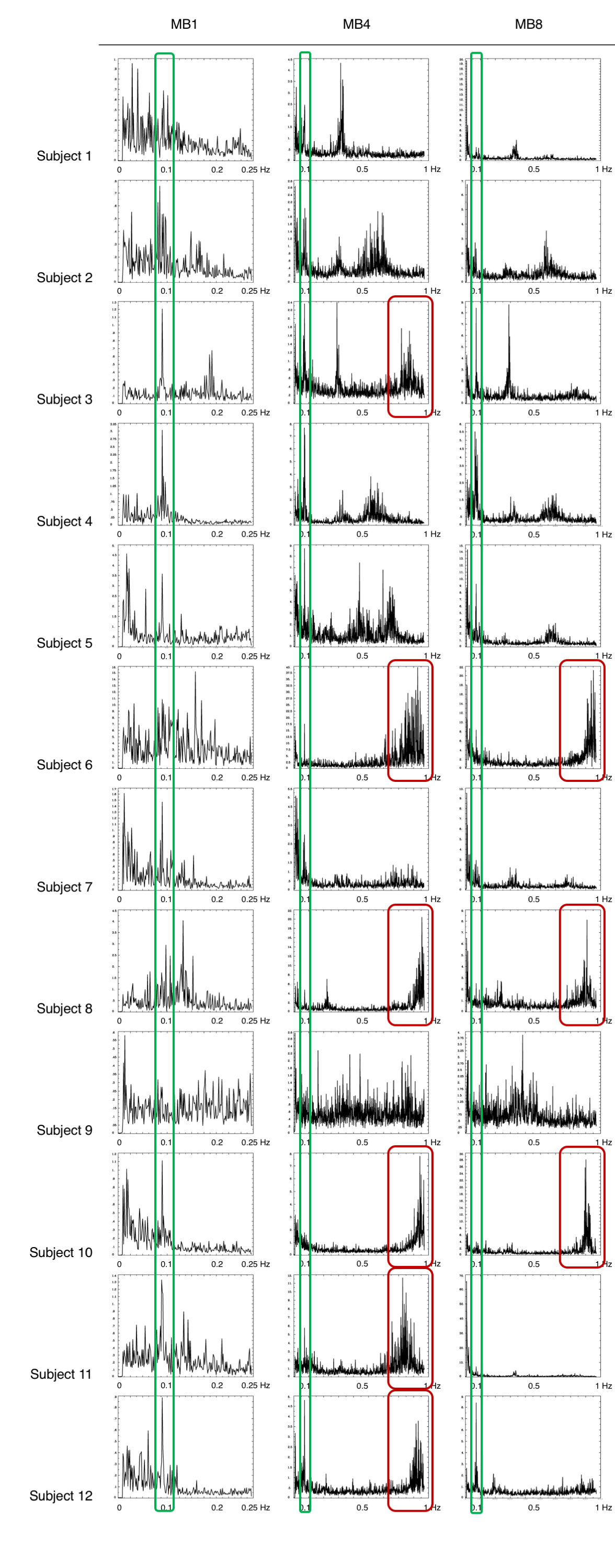


Figure S2. Smoothing Nucleus Accumbens activity time courses with low-pass filtering (at 0.25, 0.20, or 0.15 Hz).


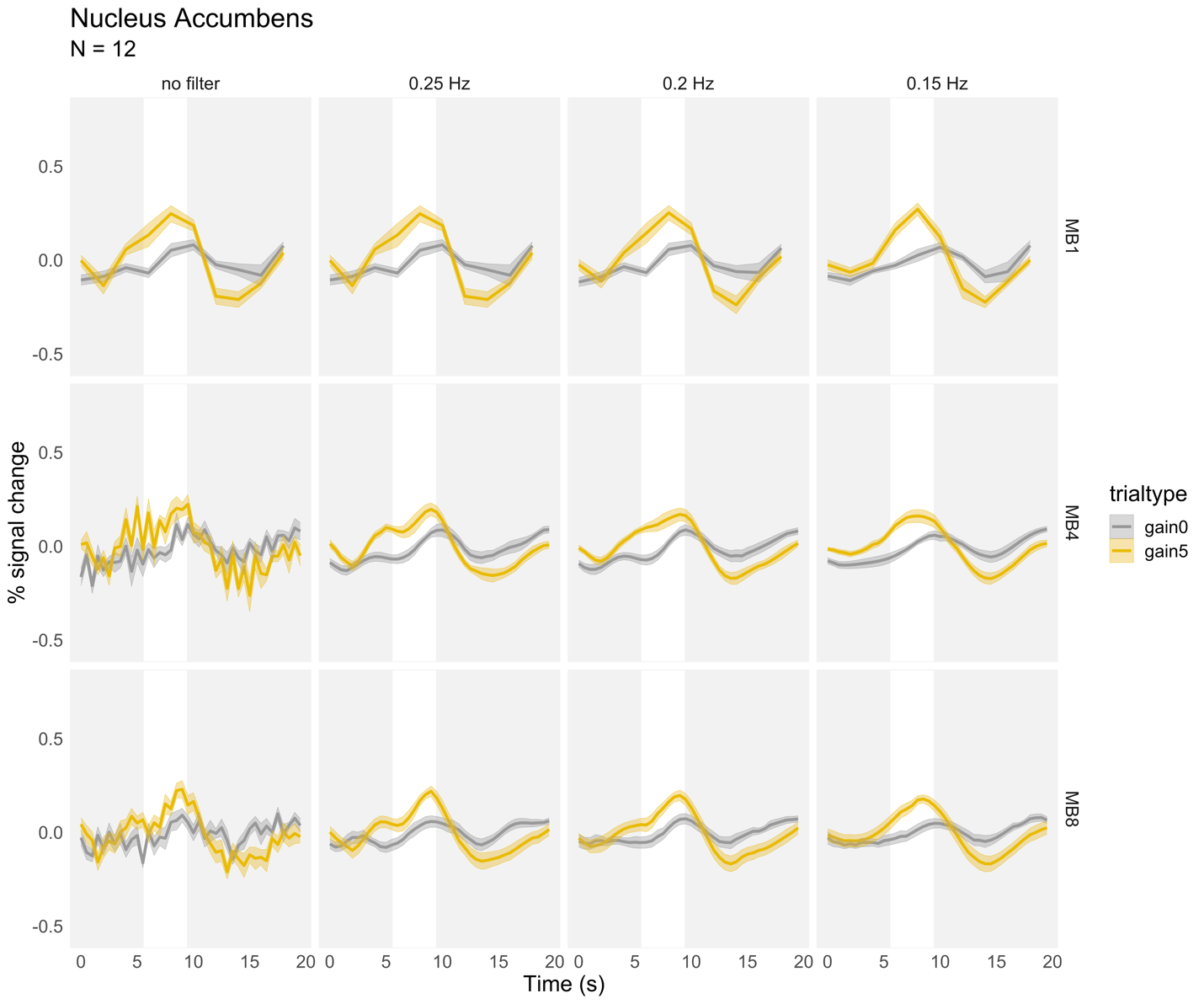


Figure S3. Low-pass filtering does not rescue multi-band induced reductions in effect size. (A) Effect size estimates (Cohen’s d ± standard error) were separately calculated for large gain versus nongain anticipation or outcome (only in MPFC) contrasts in each VOI (* *p<*0*.*05; ***p<*0*.*01; ****p<*0*.*001); (B) Lowpass filtering did not significantly influence effect size estimates in any of the VOIs, but scan type showed main effects on effect size in NAcc and V1 VOIs.


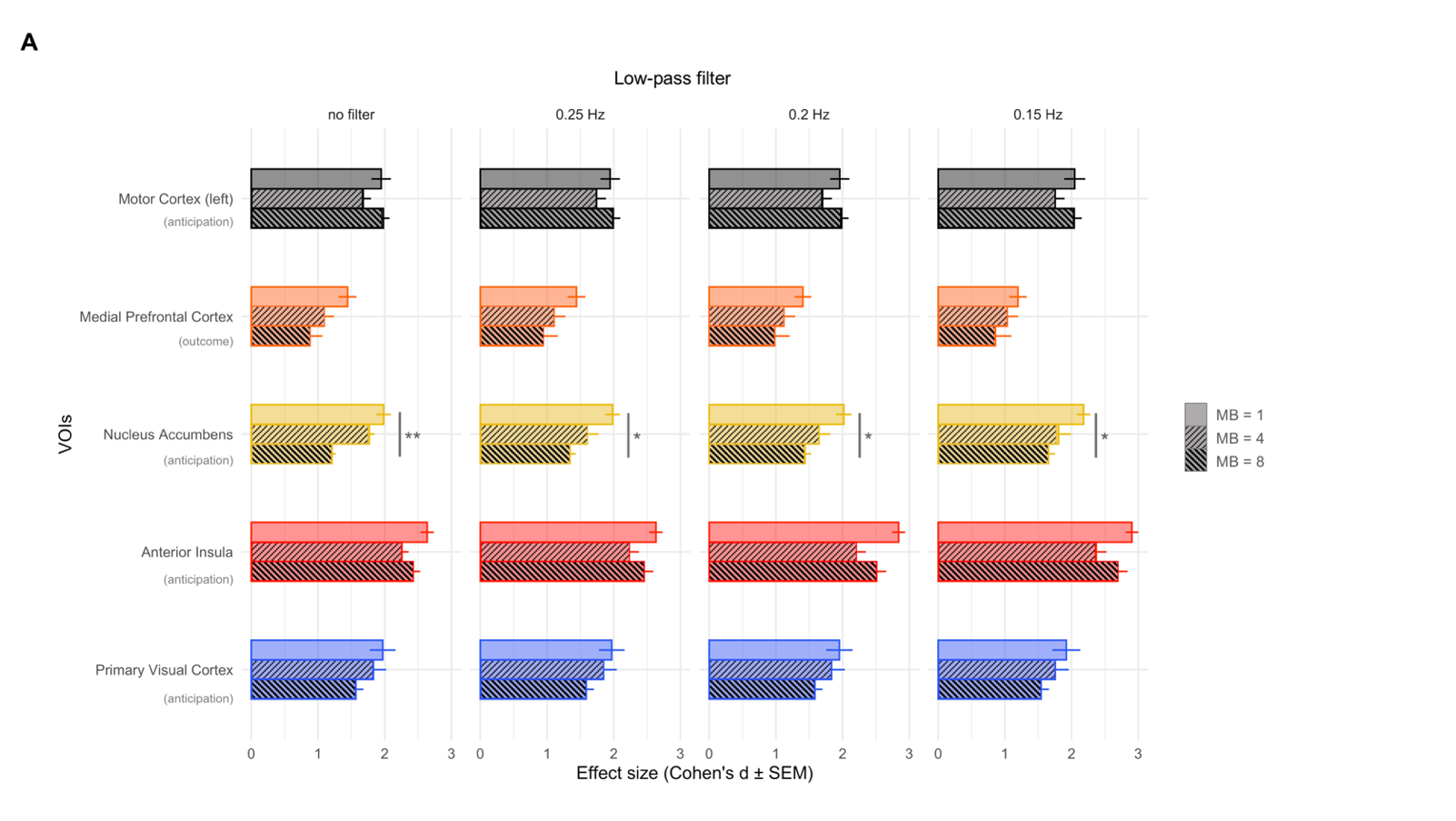


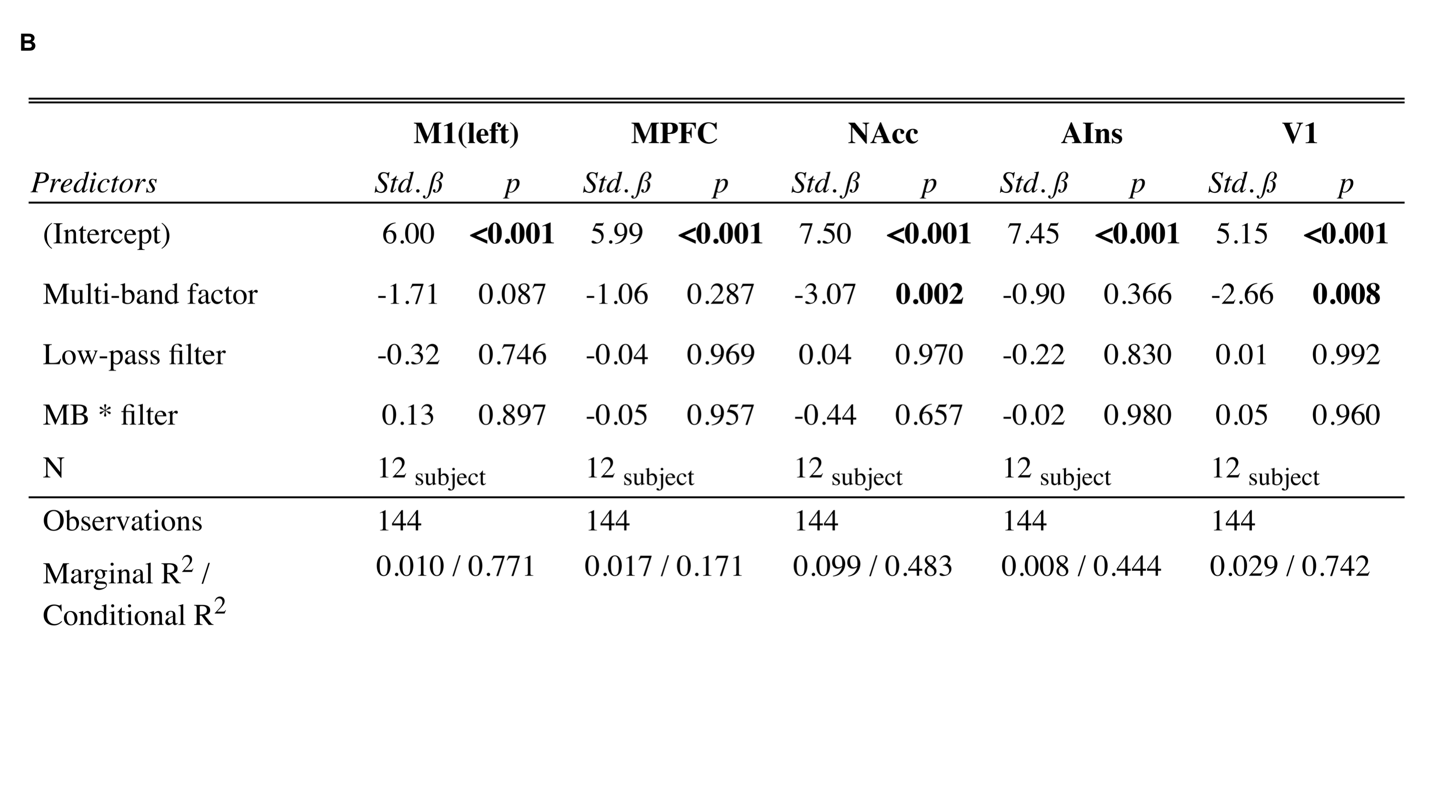


Figure S4. Whole-brain map for TSNR per unit time. TSNR is calculated as the mean over standard deviation of the resampled timeseries over the entire scan. Resampling involved averaging four volumes of multi-band data (acquired every 0.5s) to approximate the single-band sampling rate (2s).


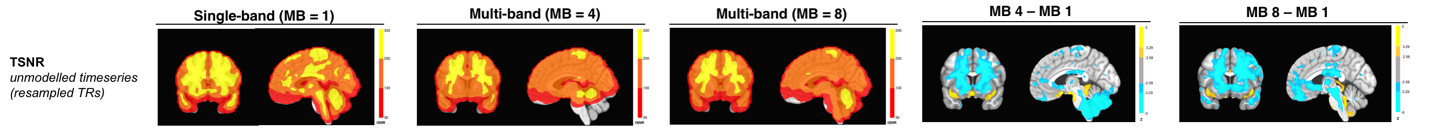


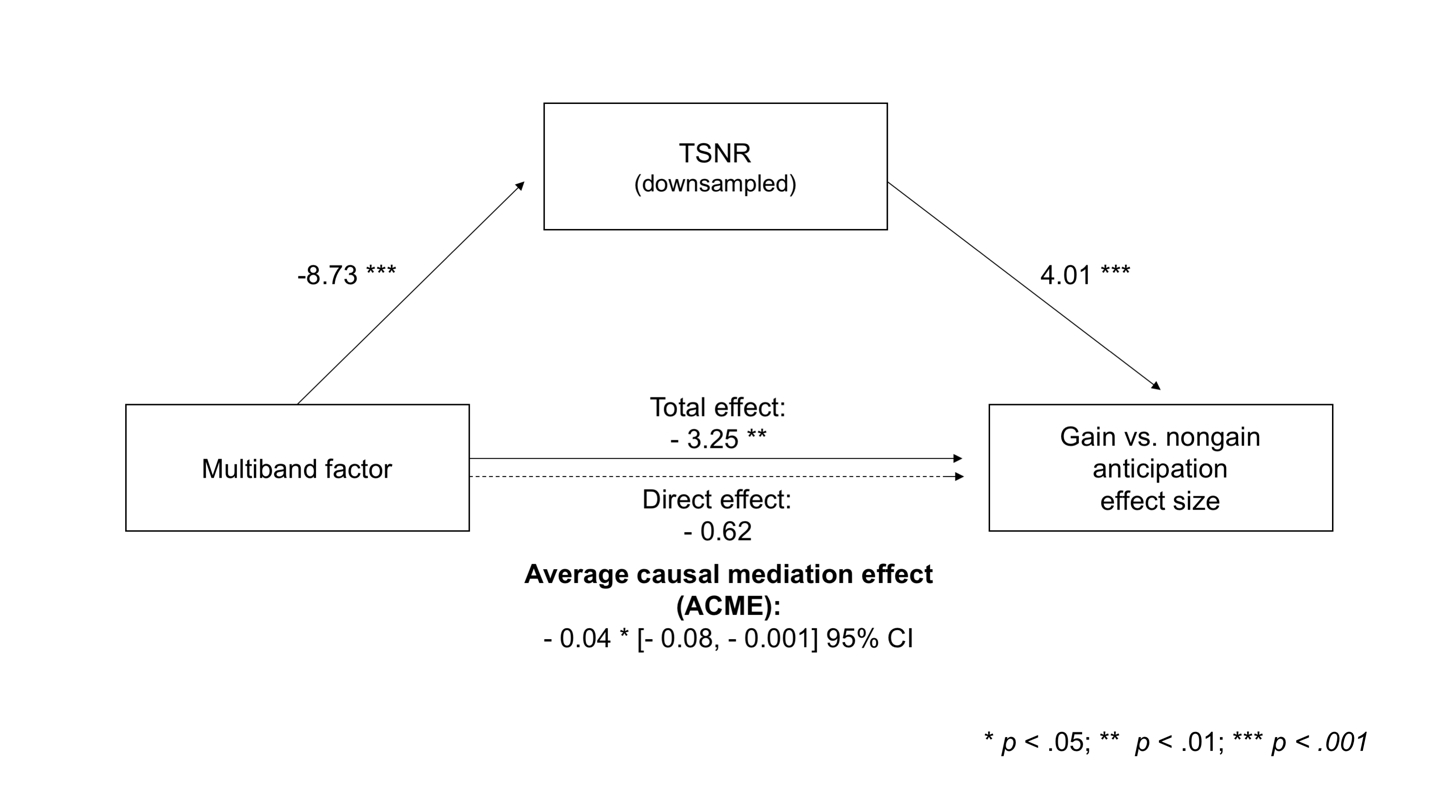
Figure S5. Downsampled TSNR statistically mediates the effect of multi-band factor on gain vs. nongain anticipation effect size in the Nucleus Accumbens.

**Supplemental References**:

Alves, F. da S., Schmitz, N., Figee, M., Abeling, N., Hasler, G., Meer, J. van der, Nederveen, A., Haan, L. de, Linszen, D., Amelsvoort, T. van, 2010. Dopaminergic modulation of the human reward system: a placebo-controlled dopamine depletion fMRI study: http://dx.doi.org/10.1177/0269881110367731 25, 538–549. https://doi.org/10.1177/0269881110367731

Andrews, M.M., Meda, S.A., Thomas, A.D., Potenza, M.N., Krystal, J.H., Worhunsky, P., Stevens, M.C., O’Malley, S., Book, G.A., Reynolds, B., Pearlson, G.D., 2011. Individuals Family History Positive for Alcoholism Show Functional Magnetic Resonance Imaging Differences in Reward Sensitivity That Are Related to Impulsivity Factors. Biol. Psychiatry 69, 675–683. https://doi.org/10.1016/J.BIOPSYCH.2010.09.049

Balodis, I.M., Kober, H., Worhunsky, P.D., Stevens, M.C., Pearlson, G.D., Potenza, M.N., 2012. Diminished frontostriatal activity during processing of monetary rewards and losses in pathological gambling. Biol. Psychiatry 71, 749–757. https://doi.org/10.1016/j.biopsych.2012.01.006

Behan, B., Stone, A., Garavan, H., 2015. Right prefrontal and ventral striatum interactions underlying impulsive choice and impulsive responding. Hum. Brain Mapp. 36, 187–198. https://doi.org/10.1002/hbm.22621

Berghorst, L.H., Kumar, P., Greve, D.N., Deckersbach, T., Ongur, D., Dutra, S.J., Pizzagalli, D.A., 2016. Stress and reward processing in bipolar disorder: a functional magnetic resonance imaging study. Bipolar Disord. 18, 602–611. https://doi.org/10.1111/bdi.12444

Boecker, R., Holz, N.E., Buchmann, A.F., Blomeyer, D., Plichta, M.M., Wolf, I., Baumeister, S., Meyer-Lindenberg, A., Banaschewski, T., Brandeis, D., Laucht, M., 2014. Impact of early life adversity on reward processing in young adults: EEG-fMRI results from a prospective study over 25 years. PLoS One 9, 1–13. https://doi.org/10.1371/journal.pone.0104185

Boukezzi, S., Baunez, C., Rousseau, P.F., Warrot, D., Silva, C., Guyon, V., Zendjidjian, X., Nicolas, F., Guedj, E., Nazarian, B., Trousselard, M., Chaminade, T., Khalfa, S., 2020. Posttraumatic Stress Disorder is associated with altered reward mechanisms during the anticipation and the outcome of monetary incentive cues. NeuroImage Clin. 25, 102073. https://doi.org/10.1016/J.NICL.2019.102073

Büchel, C., Peters, J., Banaschewski, T., Bokde, A.L.W., Bromberg, U., Conrod, P.J., Flor, H., Papadopoulos, D., Garavan, H., Gowland, P., Heinz, A., Walter, H., Ittermann, B., Mann, K., Martinot, J.-L., Paillère-Martinot, M.-L., Nees, F., Paus, T., Pausova, Z., Poustka, L., Rietschel, M., Robbins, T.W., Smolka, M.N., Gallinat, J., Schumann, G., Knutson, B., 2017. Blunted ventral striatal responses to anticipated rewards foreshadow problematic drug use in novelty-seeking adolescents. Nat. Commun. 2017 81 8, 1–11. https://doi.org/10.1038/ncomms14140

Dillon, D.G., Bogdan, R., Fagerness, J., Holmes, A.J., Perlis, R.H., Pizzagalli, D.A., 2010. Variation in TREK1 gene linked to depression-resistant phenotype is associated with potentiated neural responses to rewards in humans. Hum. Brain Mapp. 31, 210–221. https://doi.org/10.1002/HBM.20858

Doell, K.C., Olié, E., Courtet, P., Corradi-Dell’Acqua, C., Perroud, N., Schwartz, S., 2020. Atypical processing of social anticipation and feedback in borderline personality disorder. NeuroImage Clin. 25, 102126. https://doi.org/10.1016/j.nicl.2019.102126

Dutra, S.J., Man, V., Kober, H., Cunningham, W.A., Gruber, J., 2017. Disrupted cortico-limbic connectivity during reward processing in remitted bipolar I disorder. Bipolar Disord. 19, 661–675. https://doi.org/10.1111/bdi.12560

Edel, M.A., Enzi, B., Witthaus, H., Tegenthoff, M., Peters, S., Juckel, G., Lissek, S., 2013. Differential reward processing in subtypes of adult attention deficit hyperactivity disorder. J. Psychiatr. Res. 47, 350–356. https://doi.org/10.1016/j.jpsychires.2012.09.026

Fede, S.J., Grodin, E.N., Dean, S.F., Diazgranados, N., Momenan, R., 2019. Resting state connectivity best predicts alcohol use severity in moderate to heavy alcohol users. NeuroImage Clin. 22, 101782. https://doi.org/10.1016/J.NICL.2019.101782

Figee, M., Vink, M., De Geus, F., Vulink, N., Veltman, D.J., Westenberg, H., Denys, D., 2011. Dysfunctional Reward Circuitry in Obsessive-Compulsive Disorder. Biol. Psychiatry 69, 867–874. https://doi.org/10.1016/J.BIOPSYCH.2010.12.003

Filbey, F.M., Dunlop, J., Myers, U.S., 2013. Neural Effects of Positive and Negative Incentives during Marijuana Withdrawal. PLoS One 8. https://doi.org/10.1371/journal.pone.0061470

Funayama, T., Ikeda, Y., Tateno, A., Takahashi, H., Okubo, Y., Fukayama, H., Suzuki, H., 2014. Modafinil augments brain activation associated with reward anticipation in the nucleus accumbens. Psychopharmacology (Berl). 231, 3217–3228. https://doi.org/10.1007/s00213-014-3499-0

Grimm, O., Nägele, M., Küpper-Tetzel, L., de Greck, M., Plichta, M., Reif, A., 2020. No effect of a dopaminergic modulation fMRI task by amisulpride and L-DOPA on reward anticipation in healthy volunteers. Psychopharmacol. 2020 2385 238, 1333–1342. https://doi.org/10.1007/S00213-020-05693-8

Hägele, C., Schlagenhauf, F., Rapp, M., Sterzer, P., Beck, A., Bermpohl, F., Stoy, M., Ströhle, A., Wittchen, H.U., Dolan, R.J., Heinz, A., 2015. Dimensional psychiatry: Reward dysfunction and depressive mood across psychiatric disorders. Psychopharmacology (Berl). 232, 331–341. https://doi.org/10.1007/s00213-014-3662-7

Hanssen, E., van der Velde, J., Gromann, P.M., Shergill, S.S., de Haan, L., Bruggeman, R., Krabbendam, L., Aleman, A., van Atteveldt, N., 2015. Neural correlates of reward processing in healthy siblings of patients with schizophrenia. Front. Hum. Neurosci. 9, 1–11. https://doi.org/10.3389/fnhum.2015.00504

Held-Poschardt, D., Sterzer, P., Schlagenhauf, F., Pehrs, C., Wittmann, A., Stoy, M., Hägele, C., Knutson, B., Heinz, A., Ströhle, A., 2018. Reward and loss anticipation in panic disorder: An fMRI study. Psychiatry Res. - Neuroimaging 271, 111–117. https://doi.org/10.1016/j.pscychresns.2017.11.005

Holiga, Š., Sambataro, F., Luzy, C., Greig, G., Sarkar, N., Renken, R.J., Marsman, J.B.C., Schobel, S.A., Bertolino, A., Dukart, J., 2018. Test-retest reliability of task-based and resting-state blood oxygen level dependence and cerebral blood flow measures. PLoS One 13, 1–16. https://doi.org/10.1371/journal.pone.0206583

Jia, Z., Worhunsky, P.D., Carroll, K.M., Rounsaville, B.J., Stevens, M.C., Pearlson, G.D., Potenza, M.N., 2011. An initial study of neural responses to monetary incentives as related to treatment outcome in cocaine dependence. Biol. Psychiatry 70, 553–560. https://doi.org/10.1016/j.biopsych.2011.05.008

Joseph, J.E., Zhu, X., Lynam, D., Kelly, T.H., 2016. Modulation of meso-limbic reward processing by motivational tendencies in young adolescents and adults. Neuroimage 129, 40–54. https://doi.org/10.1016/j.neuroimage.2015.12.005

Jung, W.H., Kang, D.H., Han, J.Y., Jang, J.H., Gu, B.M., Choi, J.S., Jung, M.H., Choi, C.H., Kwon, J.S., 2011. Aberrant ventral striatal responses during incentive processing in unmedicated patients with obsessive-compulsive disorder. Acta Psychiatr. Scand. 123, 376–386. https://doi.org/10.1111/j.1600-0447.2010.01659.x

Kappel, V., Lorenz, R.C., Streifling, M., Renneberg, B., Lehmkuhl, U., Ströhle, A., Salbach-Andrae, H., Beck, A., 2014. Effect of brain structure and function on reward anticipation in children and adults with attention deficit hyperactivity disorder combined subtype. Soc. Cogn. Affect. Neurosci. 10, 945–951. https://doi.org/10.1093/scan/nsu135

Kaufmann, C., Beucke, J.C., Preuße, F., Endrass, T., Schlagenhauf, F., Heinz, A., Juckel, G., Kathmann, N., 2013. Medial prefrontal brain activation to anticipated reward and loss in obsessive-compulsive disorder. NeuroImage Clin. 2, 212–220. https://doi.org/10.1016/j.nicl.2013.01.005

Kocsel, N., Szabó, E., Galambos, A., Édes, A., Pap, D., Elliott, R., Kozák, L.R., Bagdy, G., Juhász, G., Kökönyei, G., 2017. Trait rumination influences neural correlates of the anticipation but not the consumption phase of reward processing. Front. Behav. Neurosci. 11, 1–10. https://doi.org/10.3389/fnbeh.2017.00085

Kostandyan, M., Park, H.R.P., Bundt, C., González-García, C., Wisniewski, D., Krebs, R.M., Boehler, C.N., 2020. Are all behavioral reward benefits created equally? An EEG-fMRI study. Neuroimage 215, 116829. https://doi.org/10.1016/J.NEUROIMAGE.2020.116829

Kryza-Lacombe, M., Hernandez, B., Owen, C., Reynolds, R.C., Wakschlag, L.S., Dougherty, L.R., Wiggins, J.L., 2021. Neural mechanisms of reward processing in adolescent irritability. Dev. Psychobiol. https://doi.org/10.1002/DEV.22090

Lawn, W., Hill, J., Hindocha, C., Yim, J., Yamamori, Y., Jones, G., Walker, H., Green, S.F., Wall, M.B., Howes, O.D., Curran, H.V., Freeman, T.P., Bloomfield, M.A.P., 2020. The acute effects of cannabidiol on the neural correlates of reward anticipation and feedback in healthy volunteers. J. Psychopharmacol. 34, 969–980. https://doi.org/10.1177/0269881120944148

Le, T.M., Chao, H., Levy, I., Li, C.S.R., 2020. Age-Related Changes in the Neural Processes of Reward-Directed Action and Inhibition of Action. Front. Psychol. 11, 1–14. https://doi.org/10.3389/fpsyg.2020.01121

Maresh, E.L., Allen, J.P., Coan, J.A., 2014. Increased default mode network activity in socially anxious individuals during reward processing. Biol. Mood Anxiety Disord. 4, 1–12. https://doi.org/10.1186/2045-5380-4-7

Mason, L., O’sullivan, N., Montaldi, D., Bentall, R.P., El-Deredy, W., 2014. Decision-making and trait impulsivity in bipolar disorder are associated with reduced prefrontal regulation of striatal reward valuation. Brain 137, 2346–2355. https://doi.org/10.1093/brain/awu152

Millman, Z.B., Gallagher, K., Demro, C., Schiffman, J., Reeves, G.M., Gold, J.M., Rakhshan Rouhakhtar, P.J., Fitzgerald, J., Andorko, N.D., Redman, S., Buchanan, R.W., Rowland, L.M., Waltz, J.A., 2020. Evidence of reward system dysfunction in youth at clinical high-risk for psychosis from two event-related fMRI paradigms. Schizophr. Res. 226, 111–119. https://doi.org/10.1016/J.SCHRES.2019.03.017

Mori, A., Okamoto, Y., Okada, G., Takagaki, K., Jinnin, R., Takamura, M., Kobayakawa, M., Yamawaki, S., 2016. Behavioral activation can normalize neural hypoactivation in subthreshold depression during a monetary incentive delay task. J. Affect. Disord. 189, 254–262. https://doi.org/10.1016/j.jad.2015.09.036

Patel, K.T., Stevens, M.C., Meda, S.A., Muska, C., Thomas, A.D., Potenza, M.N., Pearlson, G.D., 2013. Robust changes in reward circuitry during reward loss in current and former cocaine users during performance of a monetary incentive delay task. Biol. Psychiatry 74, 529–537. https://doi.org/10.1016/j.biopsych.2013.04.029

Richey, J.A., Rittenberg, A., Hughes, L., Damiano, C.R., Sabatino, A., Miller, S., Hanna, E., Bodfish, J.W., Dichter, G.S., 2014. Common and distinct neural features of social and non-social reward processing in autism and social anxiety disorder. Soc. Cogn. Affect. Neurosci. 9, 367–377. https://doi.org/10.1093/scan/nss146

Saji, K., Ikeda, Y., Kim, W., Shingai, Y., Tateno, A., Takahashi, H., Okubo, Y., Fukayama, H., Suzuki, H., 2013. Acute NK1 receptor antagonist administration affects reward incentive anticipation processing in healthy volunteers. Int. J. Neuropsychopharmacol. 16, 1461–1471. https://doi.org/10.1017/S1461145712001678

Schmidt, C., Skandali, N., Gleesborg, C., Kvamme, T.L., Schmidt, H., Frisch, K., Møller, A., Voon, V., 2020. The role of dopaminergic and serotonergic transmission in the processing of primary and monetary reward. Neuropsychopharmacology 45, 1490–1497. https://doi.org/10.1038/s41386-020-0702-3

Spaniol, J., Bowen, H.J., Wegier, P., Grady, C., 2015. Neural responses to monetary incentives in younger and older adults. Brain Res. 1612, 70–82. https://doi.org/10.1016/j.brainres.2014.09.063

Trost, S., Diekhof, E.K., Zvonik, K., Lewandowski, M., Usher, J., Keil, M., Zilles, D., Falkai, P., Dechent, P., Gruber, O., 2014. Disturbed anterior prefrontal control of the mesolimbic reward system and increased impulsivity in bipolar disorder. Neuropsychopharmacology 39, 1914–1923. https://doi.org/10.1038/npp.2014.39

Vaidya, J.G., Knutson, B., O’Leary, D.S., Block, R.I., Magnotta, V., 2013. Neural Sensitivity to Absolute and Relative Anticipated Reward in Adolescents. PLoS One 8. https://doi.org/10.1371/journal.pone.0058708

Van Duin, E.D.A., Goossens, L., Hernaus, D., Da Silva Alves, F., Schmitz, N., Schruers, K., Van Amelsvoort, T., 2016. Neural correlates of reward processing in adults with 22q11 deletion syndrome. J. Neurodev. Disord. 8, 1–12. https://doi.org/10.1186/s11689-016-9158-5

Van Hulst, B.M., De Zeeuw, P., Lupas, K., Bos, D.J., Neggers, S.F.W., Durston, S., 2015. Reward anticipation in ventral striatum and individual sensitivity to reward: A pilot study of a child-friendly fMRI task. PLoS One 10, 1–9. https://doi.org/10.1371/journal.pone.0142413

Volman, I., Pringle, A., Verhagen, L., Browning, M., Cowen, P.J., Harmer, C.J., 2020. Lithium modulates striatal reward anticipation and prediction error coding in healthy volunteers. Neuropsychopharmacol. 2020 462 46, 386–393. https://doi.org/10.1038/s41386-020-00895-2

Wang, K.S., Zegel, M., Molokotos, E., Moran, L. V., Olson, D.P., Pizzagalli, D.A., Janes, A.C., 2020. The acute effects of nicotine on corticostriatal responses to distinct phases of reward processing. Neuropsychopharmacology 45, 1207–1214. https://doi.org/10.1038/s41386-020-0611-5

Yip, S.W., Worhunsky, P.D., Rogers, R.D., Goodwin, G.M., 2015. Hypoactivation of the ventral and dorsal striatum during reward and loss anticipation in antipsychotic and mood stabilizer-naive bipolar disorder. Neuropsychopharmacology 40, 658–666. https://doi.org/10.1038/npp.2014.215
